# Bilateral Facial Palsy and Epstein–Barr Infection in Children: A Case Report and Literature Review

**DOI:** 10.3390/v18020176

**Published:** 2026-01-28

**Authors:** Simone Pilloni, Camilla Maria Pisa, Giulia Zambonini, Nicoletta de Paulis, Susanna Esposito, Giacomo Biasucci

**Affiliations:** 1 Pediatric Clinic, Pietro Barilla Children’s Hospital, Department of Medicine and Surgery, University Hospital of Parma, 43126 Parma, Italy; simone.pilloni@unipr.it (S.P.); camillamaria.pisa@unipr.it (C.M.P.); 2 Department of Medicine and Surgery, University of Parma, 43126 Parma, Italy; giacomo.biasucci@unipr.it; 3 Otorhinolaringology Unit, Guglielmo da Saliceto Hospital, 29121 Piacenza, Italy; g.zambonini@ausl.pc.it; 4 Pediatrics and Neonatology Unit, Guglielmo da Saliceto Hospital, 29121 Piacenza, Italy; n.depaulis@ausl.pc.it

**Keywords:** bilateral facial nerve palsy, Epstein–Barr virus, facial paralysis, infectious mononucleosis, Bell’s palsy, neuroinfectious disease

## Abstract

Background: Bilateral facial nerve palsy (BFNP) is a rare clinical entity in children and is more often associated with systemic or infectious diseases than unilateral facial palsy. Epstein–Barr virus (EBV) infection is an uncommon but recognized cause of facial nerve palsy and may present with bilateral involvement. Case presentation: We report the case of a 3-year-old boy who presented with progressive bilateral facial weakness following a febrile illness with pharyngitis and cervical lymphadenopathy. Neurological examination revealed complete bilateral facial paralysis (House–Brackmann grade VI). Laboratory investigations showed lymphocytosis and confirmed acute EBV infection through positive viral capsid antigen IgM and detectable EBV DNA in peripheral blood. Cerebrospinal fluid analysis demonstrated mild pleocytosis with negative EBV DNA. Brain magnetic resonance imaging revealed unilateral enhancement of the left facial nerve. Audiologic evaluation supported peripheral facial nerve dysfunction. The patient was treated with systemic corticosteroids, vitamin B complex supplementation, artificial tears, and speech therapy, resulting in gradual and substantial clinical improvement over five months. Discussion: A review of the pediatric literature identified only six previously reported cases of EBV-associated BFNP. The pathogenesis may involve either direct viral neurotropism or a post-infectious immune-mediated mechanism. Diagnostic evaluation is essential to exclude other serious causes of BFNP, particularly Lyme disease and Guillain–Barré syndrome. Conclusions: EBV infection should be considered in the differential diagnosis of BFNP in children. Prognosis is generally favorable, although recovery may be prolonged. Further studies are needed to clarify optimal diagnostic and therapeutic approaches.

## 1. Introduction

Bell’s palsy is defined as an acute, rapidly progressive unilateral facial nerve paresis or paralysis of unknown etiology, leading to partial or complete impairment of voluntary facial muscle movement [1]. Although most cases of facial nerve palsy (FNP) are idiopathic, acquired causes play a relevant role in the pediatric population, particularly infectious conditions [2]. Among these, acute otitis media and Lyme disease are well-recognized etiologies, the latter accounting for a substantial proportion of pediatric cases in endemic areas [2,3,4,5].

Bilateral facial nerve palsy (BFNP) is rare, representing only 0.3–2% of all FNP cases [6]. In contrast to unilateral facial palsy, BFNP is less frequently idiopathic and more often reflects an underlying systemic disorder, thus requiring an extensive diagnostic evaluation [7]. The most commonly associated conditions include Guillain–Barré syndrome and infectious diseases such as Lyme disease, Herpes Simplex Virus, Varicella Zoster Virus, Cytomegalovirus, and Human Immunodeficiency Virus infections [8].

Epstein–Barr virus (EBV) infection is an uncommon but increasingly recognized cause of BFNP [9]. Primary EBV infection typically occurs in early childhood and is frequently asymptomatic, while a second incidence peak in adolescence is more often associated with infectious mononucleosis [10]. Neurological complications are reported in approximately 1–5% of EBV infections and may include cranial nerve palsies, meningoencephalitis, mononeuritis multiplex, and Guillain–Barré syndrome (GBS) [10]. Notably, in pediatric patients, neurological involvement may represent the sole manifestation of EBV infection. Among reported cases of EBV-associated FNP, bilateral involvement appears relatively frequent, occurring in up to 40% of patients [11].

Given the rarity of EBV-associated BFNP in children and the limited number of detailed pediatric reports, the clinical spectrum, pathophysiology, and optimal management of this condition remain poorly defined. The present manuscript describes the case of a 3-year-old boy with BFNP secondary to acute EBV infection and provides a focused review of the pediatric literature, aiming to highlight diagnostic challenges, differential diagnoses, and therapeutic considerations. A literature search was performed to identify previously reported pediatric cases of bilateral facial nerve palsy associated with Epstein–Barr virus infection. The search was conducted using the PubMed/MEDLINE database, covering articles published up to [month year]. The following keywords and combinations were used: “Epstein–Barr virus,” “EBV,” “bilateral facial nerve palsy,” “facial diplegia,” and “children” or “pediatric.” Reference lists of relevant articles were also manually reviewed to identify additional reports. Only articles describing pediatric patients with clinical and/or laboratory evidence of EBV infection and bilateral facial nerve involvement were included.

## 2. Case Presentation

A 3-year-and-7-month-old boy with no significant past medical history presented with a 10-day history of fever, sore throat, and non-suppurative bilateral cervical lymphadenopathy. The patient was of Caucasian race and Italian ethnicity. He was initially treated with oral amoxicillin by his family pediatrician for suspected streptococcal pharyngitis. Due to progressive worsening of symptoms, including a gradual inability to close his mouth, the patient was brought to the Emergency Department. On admission, blood tests were performed, including serologic testing for the most common infectious causes of pharyngitis, which revealed acute EBV infection.

Nine days later, the child returned for further evaluation because of persistent feeding difficulties and an inability to maintain oral closure. Neurological examination revealed an amimic face, characterized by incomplete eyelid closure, absence of forehead and eyebrow movements, and loss of nasolabial folds bilaterally. BFNP was diagnosed and classified as grade VI according to the House–Brackmann grading system [12]. Grade VI according to the House–Brackmann grading system indicates complete facial paralysis, characterized by the absence of voluntary facial movement, including no forehead motion, inability to close the eyes, and no movement of the mouth on the affected side [12].

No motor or sensory deficits were observed in the limbs, and the remainder of the neurological and general physical examination was unremarkable. Inspection of the oral cavity showed resolution of tonsillar exudate, although the tonsils remained symmetrically enlarged. Painful non-suppurative bilateral cervical lymphadenopathy persisted. Lingual, palatal, and laryngeal motility were normal. Otoscopic examination revealed no clear skin or middle-ear pathology; the tympanic membranes appeared mildly hyperemic and slightly opaque bilaterally.

Laboratory investigations demonstrated leukocytosis, with a white blood cell count of 17.17 × 10^3^/μL, associated with lymphocytosis (10.08 × 10^3^/μL) and monocytosis (1.77 × 10^3^/μL). Lactate dehydrogenase (LDH) levels were elevated (322 U/L), while C-reactive protein was low (0.18 mg/dL). Liver and kidney function tests, as well as serum electrolyte levels, were within normal ranges.

Serologic testing and quantitative polymerase chain reaction (PCR) confirmed acute EBV infection, with positive antibodies against the EBV early antigen (EA IgG), positive viral capsid antigen (VCA) IgM, negative VCA IgG, negative IgG against the EBV Nuclear Antigen 1 (EBNA-1), and detectable EBV DNA in peripheral blood (11,972 cp/mL). Serologic assays and PCR panels for other neurotropic pathogens, including *Borrelia burgdorferi*, HSV, CMV, and enteroviruses, were negative.

Cerebrospinal fluid (CSF) analysis revealed mild pleocytosis (12 mononuclear cells/μL) with normal protein and glucose concentrations. EBV DNA was undetectable in the CSF, and testing for common meningoencephalitis-associated pathogens was negative.

**Figure 1:**
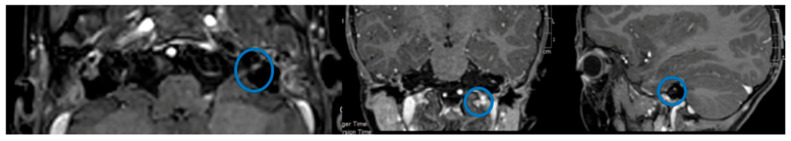
T1-weighted post-contrast sequence: minimal enhancement at the fundus of the internal auditory canal.

Brain magnetic resonance imaging (MRI) was performed two days after the diagnosis of BFNP to evaluate potential central or structural causes and to assess facial nerve involvement. The examination included contrast-enhanced T1-weighted sequences with coverage of the cerebellopontine angles, internal auditory canals, and temporal bone regions. Although a dedicated thin-slice facial nerve protocol was not available, post-contrast images were carefully reviewed along the expected anatomical course of both facial nerves. At this early stage of the disease, bilateral enhancement could theoretically be present; however, only mild enhancement of the left facial nerve at the level of the internal auditory canal and geniculate ganglion was observed (Figure 1). No parenchymal abnormalities were observed, the ventricular system was normal, and there were no signs of increased intracranial pressure or midline shift.

Audiological assessment was performed to evaluate possible involvement of the auditory system and to help localize the site of facial nerve dysfunction, given the close anatomical and functional relationship between the facial nerve and the auditory structures within the temporal bone. Audiologic assessment revealed mild low-frequency conductive hearing loss on the right side, attributed by otorhinolaryngology (ENT) specialists to middle-ear effusion. Tympanometry showed a type A curve on the right side, indicating normal middle-ear pressure and compliance, consistent with an intact tympanic membrane and normal ossicular chain mobility. In contrast, a type B curve was observed on the left side, characterized by a flat tracing, which is suggestive of middle-ear effusion or significantly reduced tympanic membrane mobility. Although otoscopic findings were not diagnostic, ENT evaluation confirmed BFNP based on the bilateral absence of the stapedial reflex, documented by impedance audiometry and video recording.

Regarding the exclusion of GBS, a careful and repeated neurological assessment was performed throughout hospitalization. The patient showed normal muscle strength in all four limbs, preserved and symmetrical deep tendon reflexes, normal gait for age, and no sensory abnormalities. No autonomic dysfunction was observed, including fluctuations in heart rate or blood pressure, respiratory compromise, or bladder or bowel dysfunction. CSF analysis demonstrated only mild pleocytosis with normal protein levels, without albuminocytologic dissociation, which is typically associated with GBS. In the absence of progressive weakness, areflexia, sensory involvement, autonomic symptoms, or clinical deterioration, neurophysiological studies were not deemed necessary. Taken together, these findings made the diagnosis of GBS unlikely in this patient.

Treatment was initiated with intravenous methylprednisolone at a dose of 30 mg/day (1.8 mg/kg/day), along with vitamin B complex supplementation and artificial tears in absence of eye protection. Logopedic therapy focusing on facial motor exercises was also started. The patient was discharged nine days after admission, and oral corticosteroid therapy was continued with prednisone at 30 mg/day, followed by a gradual weekly taper over four weeks. Gastroprotection with lansoprazole 15 mg/day was administered throughout corticosteroid treatment.

The patient remained afebrile during hospitalization, with no additional systemic symptoms. Despite persistent bilateral facial weakness, oral intake and hydration remained adequate, and feeding difficulties gradually improved without complications.

One week after treatment initiation, repeat impedance audiometry showed improvement in tympanometric findings (type As bilaterally); however, the stapedial reflex was present only on the right side at high stimulation intensities. After two weeks, the stapedial reflex could also be elicited on the left. Facial motor function improved gradually, beginning with reduced mouth opening and improved lower lip control, followed by improved eyelid closure and the appearance of a slight frontal wrinkle during attempted frowning. The child developed compensatory strategies to manage oral incompetence, such as manually closing the mouth, maintaining closure by suction, and avoiding pronunciation of bilabial consonants (“b,” “m,” and “p”).

At the conclusion of the initial course of oral corticosteroid therapy, a pediatric neurological follow-up was performed. Examination revealed a mobile head, non-painful but mildly tender on passive mobilization. The child was able to track objects in all directions of gaze but demonstrated incomplete bilateral eyelid closure on command. Facial asymmetry was mild, with persistent partial mouth opening and symmetric flattening of the nasolabial folds, more pronounced on the left. Tongue and palate mobility were normal. A partial bilateral deficit of the frontalis muscle persisted, along with marked dysfunction of the orbicularis oris muscle. The patient was only partially able to move the mouth and inflate the cheeks and continued to experience articulation difficulties. Facial nerve function was graded as House–Brackmann grade IV [12]. House–Brackmann grade IV corresponds to moderately severe facial nerve dysfunction, characterized by obvious facial asymmetry at rest, incomplete eye closure, and limited but present voluntary facial movement [12]. A second course of corticosteroid therapy was prescribed for four weeks, starting at 1 mg/kg/day for one week, followed by gradual weekly tapering. A second course of corticosteroid therapy was initiated due to incomplete recovery of facial nerve function after the initial treatment, with persistent moderately severe bilateral facial weakness (House–Brackmann grade IV). The aim was to further reduce ongoing inflammation, limit immune-mediated nerve injury, and promote continued functional recovery of the facial nerve.

At a subsequent neurological follow-up one month later, further clinical improvement was observed. Ocular motility was normal, and eyelid closure was complete bilaterally, with only minimal residual asymmetry on the left. Facial symmetry was largely restored, with the mouth predominantly closed and only subtle flattening of the left nasolabial fold. Tongue and palate movements remained normal. A mild bilateral deficit of the frontalis muscle persisted. Function of the orbicularis oris had significantly improved, allowing effective mouth movements and stretching of the oral commissures, with minimal residual asymmetry (right > left). The child was able to inflate his cheeks, and articulation of phonemes had noticeably improved. Overall, these findings indicated a substantial recovery of bilateral facial nerve function.

Table 1 summarizes clinical course and main neurological and ENT findings from admission to the latest follow-up.

## 3. Discussion

To our knowledge, only six cases of BFNP associated with EBV infection in children have been reported prior to our case. The key characteristics of these cases are summarized in Table 2 [3,8,11,13,14,15].

The age range of patients in these reports, including our own case, spans from 14 months to 18 years, indicating that BFNP due to EBV can occur across the entire pediatric age group. Notably, no clear gender differences were observed, with four males and three females affected.

As previously mentioned, FNP due to EBV infection has been documented, although its exact incidence remains unclear. The predisposing factors that might induce FNP in the context of EBV infection are not fully understood, though otitis media is one potential contributing factor. The etiological mechanisms proposed in the literature generally involve either direct viral invasion of the facial nerve or a post-infectious immune-mediated inflammatory response within the CNS [11]. In our case, it is difficult to ascertain which of these mechanisms predominates, given that the child presented with mononucleosis-like symptoms prior to developing BFNP, which suggests a post-infectious mechanism. However, the presence of middle ear effusion and non-suppurative reactive cervical adenopathy observed during the ENT examination points to an ongoing infectious process. The absence of other systemic symptoms of infectious mononucleosis during the hospital stay, despite the persistence of BFNP, may further support the hypothesis of a post-infectious inflammatory response. Although the detection of EBV DNA in peripheral blood together with VCA IgM positivity strongly supports the diagnosis of acute EBV infection, the pathogenic mechanism underlying BFNP remains incompletely understood and largely speculative. In our patient, the absence of EBV DNA in the cerebrospinal fluid, the delayed onset of neurological symptoms following systemic signs of infection, and the favorable response to corticosteroid therapy argue against direct viral invasion and instead support a post-infectious, immune-mediated mechanism. Nevertheless, concomitant local inflammatory factors, such as middle-ear effusion and regional lymphadenopathy, may have contributed to facial nerve involvement by increasing nerve vulnerability within the facial canal. Given the rarity of EBV-associated BFNP and the limited number of reported pediatric cases, definitive conclusions regarding pathogenesis cannot be drawn, underscoring the need for further studies to clarify the mechanisms linking acute EBV infection and facial nerve dysfunction.

One of the most intriguing aspects of our case is the bilateral involvement of the facial nerves, which remains a rare phenomenon in FNP cases, as previously noted. Interestingly, among FNP cases attributed to EBV infection, up to 35% are bilateral, as suggested by the literature [13]. The reason for this relatively high incidence of BFNP in EBV-associated FNP is still not well understood. A contributing factor could be otomastoiditis, as identified in two of the cases reviewed by Terada et al. [13]. However, it is important to note that BFNP is not a common feature of bilateral otomastoiditis in the absence of EBV infection [13].

The diagnosis of BFNP in our case was supported by the absence of the stapedial reflex bilaterally, as confirmed by impedance audiometry. This electrophysiological finding is consistent with peripheral facial nerve dysfunction.

MRI results were particularly interesting, as they revealed inflammatory involvement of the facial nerve only on the left side, with no similar alterations on the right side. This finding contrasts with two other cases in the literature, where bilateral facial nerve involvement was noted on MRI [8,11]. The discrepancy between our MRI findings and those in the literature may be attributed to differences in the timing of clinical presentation versus radiological imaging, which could partially explain the unilateral involvement seen in our patient. The absence of enhancement on the right side may reflect temporal variability in inflammatory changes, differences in disease severity, or limitations related to slice thickness and spatial resolution. These considerations underscore that MRI findings in facial nerve palsy must be interpreted in conjunction with clinical and electrophysiological data and that a lack of bilateral enhancement does not exclude bilateral facial nerve involvement.

Regarding the diagnosis of EBV infection as the cause of FNP, historical diagnostic methods have evolved over time. Prior to the 1970s, diagnosis was based primarily on clinical symptoms, detection of atypical lymphocytosis in peripheral blood, and the presence of heterophile antibodies. For example, heterophile antibodies were used for diagnostic confirmation in the cases reported by Egan [14] and Weintraub [15,16], although Weintraub also measured EBV serum titers. In contrast, the detection of EBV DNA in peripheral blood has become a more reliable and early diagnostic marker of primary EBV infection, often correlating with clinical manifestations of the disease [11]. In our case, the detection of EBV DNA in peripheral blood supported the diagnosis of an acute phase EBV infection. This finding was also observed in the reports by Weintraub [15,16], Terada [13], and Messana [11].

Serologic testing for EBV typically involves measuring IgG against EA, IgM and IgG against the VCA, and IgG against the EBNA-1, which are considered the most specific markers for EBV infection [10]. In all the reported cases, except for the earlier studies by Egan [14] and Weintraub [15,16], these serological markers were used to confirm EBV infection, reflecting the advancement in diagnostic techniques over time. Interestingly, EBV-induced polyclonal B lymphocyte activation can interfere with serologic tests for other infectious diseases, such as Lyme disease, by cross-reacting with antibodies against *Borrelia burgdorferi* [17]. In our patient, serologic testing for *Borrelia burgdorferi* was negative, which is crucial because FNP can also present in the context of Lyme disease. Although the incidence of Lyme borreliosis in Italy shows marked geographic variability [18], a careful clinical history revealed no known tick exposure, no history of erythema migrans, and no recent travel to highly endemic areas. Serologic testing for *Borrelia burgdorferi* was performed using a standard two-tier approach, including enzyme immunoassay followed by confirmatory immunoblot, and results were negative [18]. In the absence of epidemiological risk factors, characteristic clinical features, or laboratory evidence of infection, Lyme disease was considered unlikely in this patient. Early detection and appropriate treatment are essential to prevent potential complications of Lyme disease.

The role of cerebrospinal fluid (CSF) analysis in diagnosing EBV-related FNP is noteworthy. EBV DNA is typically absent in the CSF of patients with neurological complications of EBV infection, including FNP [8,11]. In our case, CSF analysis revealed only mild pleocytosis (12 mononuclear cells/μL), and EBV DNA was undetectable, which aligns with findings from other case reports.

Therapeutic approaches for EBV-related FNP vary widely. Although EBV infection is self-limiting, certain treatments may be employed to alleviate symptoms. Acyclovir, which inhibits viral replication [19], has been used in some cases, although there is no evidence to recommend routine use of acyclovir alongside corticosteroids in EBV infections [19]. In our case, we opted to treat with oral prednisone (1.8 mg/kg/day), gradually tapering over one month. Steroids are commonly used to reduce inflammation and shorten the duration of symptoms in complicated EBV infections. Additionally, although there is no evidence on its efficacy, we administered vitamin B complex supplementation to support nerve function recovery and artificial tears to address corneal exposure due to incomplete eyelid closure. Logopedic therapy, focusing on facial motor exercises, was also initiated to promote facial muscle recovery. Literature reports vary in their therapeutic strategies. Acyclovir was used by Terada et al. [13] and Forci et al. [8], while steroids (prednisone or prednisolone) were administered in all cases except for Egan’s report [14]. The duration of steroid therapy ranged from 7 days in Terada et al.’s report [13] to 6 weeks in Messana et al.’s case [11].

Recovery from FNP due to EBV varies widely across reported cases, with resolution times ranging from 1 month (Forci et al. [8]) to 9 months (Messana et al. [11]). In our case, the resolution occurred within 2 months, which is consistent with the literature indicating a generally good prognosis for EBV-associated FNP, though recovery timelines can vary depending on factors such as the severity of the initial clinical presentation and the timing of treatment initiation. Recovery typically progresses from eye closure to improvement in mouth and facial expressions [11].

## 4. Conclusions

BFNP is a rare but clinically significant condition in children and should prompt careful evaluation to exclude serious systemic and infectious causes, particularly GBS and Lyme disease. Although uncommon, EBV infection should be considered among the potential etiologies, even in the absence of EBV DNA in the CSF, especially when supported by compatible serologic findings.

Management of EBV-associated BFNP is mainly supportive and aimed at preventing ocular, respiratory, and feeding complications while promoting functional recovery. Corticosteroids are frequently used in clinical practice, although evidence-based treatment recommendations are lacking. Overall, the prognosis in pediatric patients appears favorable, with gradual recovery occurring over weeks to months.

This report is limited by its descriptive nature as a single case and by the absence of standardized diagnostic and therapeutic protocols for EBV-related BFNP. Larger studies and systematic analyses are needed to better clarify the underlying pathophysiological mechanisms and to define optimal management strategies for this rare condition.

## Figures and Tables

**Table 1:** Clinical course and main neurological and ENT findings from admission to the latest follow-up.

Date	Clinical Status	Neurological Findings	ENT/Ophthalmologic Findings
Admission	Good general condition, afebrile, alert; facial amimia; feeding preserved	Bilateral facial weakness; inability to close mouth/eyes; no limb deficits	Bilateral facial immobility
After 2 days	Stable	Bilateral facial nerve palsy (HB VI); normal strength, reflexes, gait; mild speech articulation difficulty	Mild right low-frequency conductive hearing loss; tympanogram As (R), B (L)
After 5 days	Stable	Facial motor function unchanged	Persistent bilateral facial palsy (HB VI); minimal lower lip movement
After a week (discharge)	Good general condition; normal feeding and hydration	Persistent bilateral facial palsy (HB VI) with minimal improvement	Stable ENT findings; compensatory facial movements
After one month	Stable	Mild improvement in orofacial motor function	Speech therapy: improving praxies
After three months	Improving	Progressive recovery of facial motility	Speech therapy: continued improvement
After five months	Good general condition	Improved facial motility; no other deficits	No relevant ENT findings

**Table 2:** Summary of the main characteristics of cases of bilateral facial palsy due to EBV infection in children reported in the literature.

Age (Gender)	Mode of Diagnosis	MRI Findings	Therapy	Time from Onset of Palsy to Full Recovery	Author, Year (Reference)
18 years (M)	Clinical, heterophile-antibody agglutination	//	High caloric diet, multivitamins, facial physiotherapy	Only partial resolution after 4 months	Egan, 1960 [14]
15 years (F)	Clinical, heterophile-antibody, serum EBV titers	//	Prednisone (dose and duration not specified)	Few months	Weintraub, 1976 [16], Weintraub 1977 [15]
14 months (F)	Peripheral blood atypical lymphocytes, VCA IgG (after 1 month), EBNA (after 5 months), EBV DNA in peripheral blood	//	Acyclovir (15 mg/kg/day) EV, prednisolone (1 mg/kg) PO for 7 days	12 weeks	Terada et al., 2004 [13]
16 years (M)	Clinical, IgM and IgG for EBV (also IgM and IgG for *Borrelia burgdorferi*, possible cross-reaction), negative CSF for EBV	Bilateral enhancement of the 7th cranial nerve in the internal auditory canal	Systemic corticosteroids, acyclovir	1 month	Forci et al., 2017 [8]
8 years (F)	IgM and IgG for EBV (also IgM for *Borrelia burgdorferi*, not confirmed in a second control), positive blood PCR for EBV (850 copies/mL)	Bilateral inflammation of facial nerve	Prednisolone (1 mg/kg/day) for 6 weeks on scalar dosage, vitamin B complex	9 months	Messana et al., 2018 [11]
2 years (M)	Clinical, IgM anti VCA, negative CSF for EBV	Paranasal sinus disease, mastoid fluid	Oral prednisolone taper (starting from 1 mg/kg/day), bilateral tympanostomy with tube placement	3 months	Guess et al., 2020 [3]
3 years (M)	Clinical, IgM anti VCA, EBV DNA in peripheral blood, negative CSF for EBV	Mild enhancement at the left internal auditory canal along the course of the facial nerve and at the geniculate ganglion	oral prednisone with taper dosage (starting from 1.8 mg/kg/day), vitamin B complex supplementation, artificial tears, logopedic therapy focused on facial motor exercises	3 months	Our case

Abbreviations: M = male; F = female; MRI = Magnetic Resonance Imaging; EBV = Epstein–Barr Virus; VCA = Viral Capsid Antigen; EBNA = Epstein–Barr Viral Nuclear Antigen; DNA = Deoxyribonucleic Acid; EV = Endovenous; PO = Per Os; Ig = Immunoglobulin; CSF = Cerebrospinal Fluid; PCR = Polymerase Chain Reaction; // = No information reported in the article.

## Data Availability

All the available data are included in the manuscript.
